# CODA: Accurate Detection of Functional Associations between Proteins in Eukaryotic Genomes Using Domain Fusion

**DOI:** 10.1371/journal.pone.0010908

**Published:** 2010-06-01

**Authors:** Adam J. Reid, Juan A. G. Ranea, Andrew B. Clegg, Christine A. Orengo

**Affiliations:** 1 Wellcome Trust Sanger Institute, Cambridge, United Kingdom; 2 Research Department of Structural and Molecular Biology, University College London, London, United Kingdom; 3 Department of Molecular Biology and Biochemistry, University of Malaga, Malaga, Spain; University of Manchester, United Kingdom

## Abstract

**Background:**

In order to understand how biological systems function it is necessary to determine the interactions and associations between proteins. Gene fusion prediction is one approach to detection of such functional relationships. Its use is however known to be problematic in higher eukaryotic genomes due to the presence of large homologous domain families. Here we introduce CODA (Co-Occurrence of Domains Analysis), a method to predict functional associations based on the gene fusion idiom.

**Methodology/Principal Findings:**

We apply a novel scoring scheme which takes account of the genome-specific size of homologous domain families involved in fusion to improve accuracy in predicting functional associations. We show that CODA is able to accurately predict functional similarities in human with comparison to state-of-the-art methods and show that different methods can be complementary. CODA is used to produce evidence that a currently uncharacterised human protein may be involved in pathways related to depression and that another is involved in DNA replication.

**Conclusions/Significance:**

The relative performance of different gene fusion methodologies has not previously been explored. We find that they are largely complementary, with different methods being more or less appropriate in different genomes. Our method is the only one currently available for download and can be run on an arbitrary dataset by the user. The CODA software and datasets are freely available from ftp://ftp.biochem.ucl.ac.uk/pub/gene3d_data/v6.1.0/CODA/. Predictions are also available via web services from http://funcnet.eu/.

## Introduction

In the post-genomic era it has become clear that the parts list of genomes is insufficient to explain organismal complexity. Research is shifting towards understanding organisms as systems of interacting parts. Many new approaches are being developed to identify the relationships between these parts in terms of interactions and functional associations. Gene or domain fusion is one of several genome context methods which can be used to predict functional associations between pairs of proteins [1], [2]. Genome context methods allow inheritance of functional information between non-homologous proteins and are thus an orthogonal approach to homology-based methods of function prediction. In addition, they can predict networks of proteins involved in common complexes and pathways [3].

Gene fusion is an evolutionary process whereby initially separate genes become fused into a single open reading frame, which is expressed as a multi-domain protein chain. By detecting these events it can be inferred that the unfused proteins are functionally related. Bioinformatic approaches which identify fusion events in order to predict functional associations use either whole protein sequence comparison or domain family assignments. These are known as gene fusion and domain fusion respectively. The two approaches are compatible and have previously been combined [2]. Table 1 shows the various implementations of gene/domain fusion detection which have appeared in the literature. The most common approach is gene fusion detection using BLAST [4], and/or Smith-Waterman [5]. In this scheme two proteins from a single genome (query proteins), both predicted to be homologous to a third protein in a different genome (fusion protein), are identified as functionally associated (i.e. take part in a common biological process). In the case of domain fusion, protein domain family annotation (e.g. Pfam [6]) is used to identify two proteins from one genome which contain distinct domains that are found fused in another genome.

**Table 1:** Overview of gene/domain fusion implementations for predicting functional associations.

Authors	Fusion detection method	All homologues/Orthologues-only	Scoring
Marcotte et al. [2]	Gene fusion (BLAST) and domain fusion (ProDom) pooled.	All homologues – 5% most promiscuous domains removed	None
Enright et al. [1]	Gene fusion (BLAST and S-W)	All homologues	S-W based Z-scores
Snel et al. [9]	Gene fusion (S-W)	Orthologue-only (bidirectional best hit)	# fusions events/# target genomes
Enright & Ouzounis [21]	Gene fusion (BLAST, component overlap <10%)	All homologues (although component and composite proteins clustered)	None
Yanai et al. [22]	Gene fusion (BLAST)	Orthologue-only (one link between each COG)	None
Marcotte & Marcotte, [7]	Gene fusion (BLAST)	All homologues	Probability of observing fusion and uncertainty due to large families
Truong & Ikura [12]	Domain fusion (Pfam domains)	All homologues (promiscuous domains removed)	None
Bowers et al. [11]	Gene fusion (BLAST)	All homologues	Probability of observing fusion
Reid et al., this work	Domain fusion (Pfam domains)	All homologues	Frequency of homologues in query and individual target genomes

Those methods designed purely for examining gene fusion as an evolutionary process have been excluded. ‘All homologues’ refers to the approach of allowing all hits between protein pairs which are homologous to a fusion protein, while ‘orthologue-only’ refers to methods which only allow hits where the query proteins are orthologous to a fusion protein.

The principle problem in accurately detecting functional relationships with these methods is caused by large, promiscuous domain families. If a relative of domain family A is found fused to a relative from domain family B, all proteins containing domains from A are potentially associated to all those containing domains from B within any particular genome. If families A and B are large, then there are many possible functionally associated pairs. Large families tend to be functionally diverse and it is unlikely that all members will be involved in the same biological process [7].

Promiscuous domain families are found in many different proteins, fused to many different partner domain families [8]. The protein kinase domain family Pkinase (Pfam code: PF00069) is one of the most promiscuous in nature. It largely comprises eukaryotic protein kinases involved in diverse biological processes. The result of this is noise in the domain fusion analysis through functionally misinformative fusions. Any protein containing members of the Pkinase family can be linked to every other protein which contains one of the >250 domains to which Pkinase is found fused.

Large families tend to be promiscuous and vice versa, therefore solving one problem solves the other. One approach is to simply exclude proteins containing highly promiscuous domains [2]. Alternatively only those pairs of query proteins which are thought to be orthologous to the fusion protein are accepted [9]. This results in high accuracy, although relatively few functional relationships are determined – a maximum of one per fusion protein in any particular genome [10]. The third approach is to apply a scoring scheme which takes account of the size of families and the uncertainty about which pairs are orthologous [7]. Because we expect some paralogues to take part in the same biological processes, this approach allows more predictions to be made, while maintaining a reasonable degree of accuracy [7]. To our knowledge no assessment has been published of the relative performance of these solutions, or any contrasting implementations of gene/domain fusion. It is especially important to find good solutions to this problem as higher eukaryotes such as humans have more large, promiscuous domain families than other organisms.

Our method, CODA, uses the domain fusion approach and implements a novel score to cope with the problem of large, promiscuous families. CODA is compared against two existing implementations of gene fusion and one of domain fusion. STRING-fusion is a gene fusion approach used for the STRING database by the Bork group [3]. Prolinks-fusion is a gene fusion approach used in the Prolinks database by the Eisenberg group [11]. Truong-fusion is a domain fusion method employed by Truong & Ikura [12]. These respectively represent orthologue-only gene fusion, scored gene fusion and domain fusion with exclusion of promiscuous families.

We show that in several cases CODA can produce a greater number of hits than other methods while maintaining accuracy and more generally that gene/domain fusion approaches can succeed in higher eukaryotes to a similar degree as in lower eukaryotes. Furthermore we find that the different methods are complementary, with low overlap between the results they produce.

CODA is available for download and can also be used through web services, allowing users to determine functional relationships in their genome of interest.

## Results

Initially we show how different domain family classifications affect the performance of our method CODA (Co-Occurance of Domains Analysis) and how it copes with the problem of promiscuous domains. Subsequently CODA is compared to other gene/domain fusion approaches. Benchmarks were performed using a measure of similarity between the Gene Ontology (GO) terms of each functionally associated protein pair predicted in the query genome by each method. We use an ‘enrichment’ score where a score of 10 means that we have found 10 times as many functionally similar pairs of proteins as would be expected by chance.

CODA was developed using *Saccharomyces cerevisiae* (hereafter referred to as yeast) as the query genome as this genome has the most comprehensive functional annotation. The human genome is used as an example of a complex higher eukaryote and to provide an independent test of performance.

### Performance of CODA

#### Alternative domain classifications

CODA uses domain pairs to identify fusions. In previous work ProDom and Pfam domains have been used to detect domain fusions for prediction of functional associations [1], [12], [13], however structural domain representations (e.g. SCOP & CATH) have not been explored in these terms. We found that datasets based principally on CATH domains (CATH-Pfam and CATH) performed less well than those based on Pfam domains (see Supplementary Figure S1). This could have been because CATH superfamilies tend to be broader than Pfam families, including more functional subfamilies, resulting in reduced scores for hits involving these larger families. Additional analysis using CATH subfamilies did not improve performance however (see Supplementary Figure S2) and thus it is more likely that CATH had lower performance due to lower coverage of the genomes. Multi-Domain Architecture (MDA) datasets containing only Pfam domains were used for subsequent analyses.

#### CODA is insensitive to promiscuous domains

Domain fusion methods are liable to detect many false positives due to promiscuous domains and large families [7]. This problem is tackled by the CODA score which takes account of the size of domain families. As large families tend to be promiscuous, the scoring method should also penalise promiscuity. Here a promiscuous domain family is described as one which co-occurs with more than 50 other domain families.

**Figure 1:**
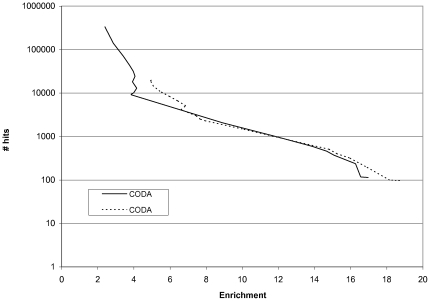
CODA with and without promiscuity filter (prom50). The promiscuity filter removes all results involving a domain that is known to occur in protein chains with 50 or more different domain families, across all genomes. Enrichment is a measure of accuracy: the number of true positives divided by the number of positives expected by chance given the number of hits (see methods).

Figure 1 shows that CODA coped well with promiscuous domains, finding a greater number of hits for an enrichment of 10 when promiscuous domains were present (1663) compared to when they were removed (1494).

### Comparison of CODA with pre-existing methods

We wanted to determine the performance of CODA relative to comparable methods. To our knowledge the relative effectiveness of different gene/domain fusion methods has been unclear. While gene fusion has the potential to exploit all known genes, domain fusion only has access to those parts of genes classified into domain families. However, because domain families are composed using powerful methods for detecting homologues (e.g. profile Hidden Markov Models), domain fusion approaches can detect more distantly related fusion events. Sequence comparison approaches (e.g. Smith-Waterman and BLAST) used in gene fusion approaches cannot detect such distant relationships. Thus it is unclear whether one approach might provide more coverage than the other.

Several resources provide functional associations derived from such methods. These include STRING [9], Prolinks [11] and the Domain Fusion Database [12]. These methods could not be run on arbitrary sets of genomes/sequences. Therefore, to benchmark CODA against these datasets, it was necessary to use only those sequences which had been used to produce the results provided by the respective web servers. Furthermore, because of this, it was not possible to directly compare all three methods. CODA was compared to STRING-Fusion on the STRING sequence set, to Prolinks-Fusion on the Prolinks sequence set, and to Truong-fusion on the Truong dataset.

#### Relative performance of CODA and other methods

**Figure 2:**
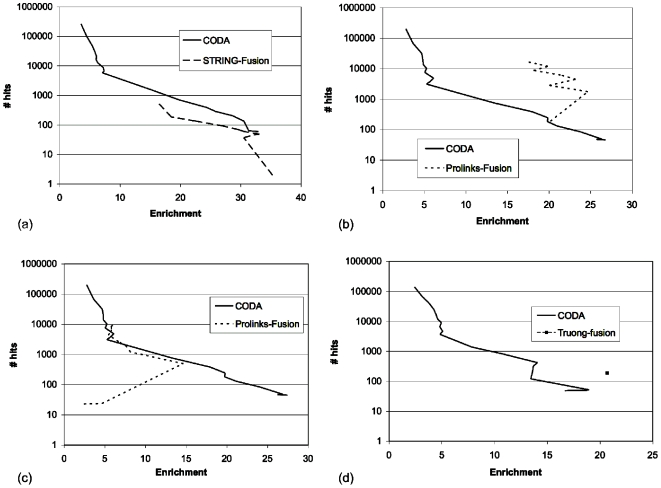
Comparisons between CODA and other methods on the yeast genome. (a) Relative performance of CODA and STRING-fusion methods on the STRING dataset. (b) Relative performance of CODA and Prolinks-fusion on the Prolinks dataset. (c) Relative performance of CODA and Prolinks-Fusion on the Prolinks dataset, with all results between homologous pairs removed (BLAST E-value <1e-6). (d) Relative performance of CODA and Truong-fusion on the Truong dataset.

Figure 2a shows that CODA outperformed STRING-Fusion at almost all levels of enrichment. STRING-Fusion considers only pairs of proteins thought to be orthologous to fusion proteins and so had a relatively small maximum number of hits, 548. This was at an enrichment of 16.3. For a similar enrichment, CODA found 1549 hits. STRING-fusion was able to achieve higher accuracy, although only finding a very small number of fusions.

Figure 2b shows that Prolinks-fusion outperformed CODA. For an enrichment of 10 CODA found 1312 protein pairs while Prolinks-fusion found 17361 pairs (all its results) for a higher enrichment of 17. Figure 2c shows that the improved performance of Prolinks over CODA was due to a large number of links between homologues. In fact when homologous pairs were removed from the results of both methods (pairs with BLAST E-value< = 1e-6), CODA found 1306 protein pairs for an enrichment of 10, while Prolinks-fusion found only 1021. Note that CODA explicitly excludes pairs with homologous domains.

Figure 2d shows the results for CODA against Truong-fusion. There is no score provided for results from Truong-fusion and so there is only one point on the graph referring to the complete set of 189 pairs of proteins identified by the method. We see that compared to CODA, Truong-fusion was more accurate for the number of hits it produces, with an enrichment of 21 for 189 hits. CODA finds 52 hits for an enrichment of 19 and was able to find 1023 hits for an enrichment of 10.

#### Domain fusion methods find functional associations for more proteins than gene fusion methods

Do fusion methods tend to find many links between few proteins, or few links between many proteins? In order to examine the number of links vs. proteins produced by the methods the first 500 top scoring hits from CODA were taken for comparison with the top 500 from STRING-fusion, as STRING-fusion only produced ∼500 hits (Supplementary Figure S3). For comparison between CODA and Prolinks-fusion the first 1000 hits were taken (Figure S4). Truong-fusion only produced 189 hits and so these were compared to the first 189 hits from CODA (Figure S5). The results show that in all cases CODA had a roughly 1∶1 relationship between new links and proteins, i.e. for each novel link, one of the proteins had not been seen before. Both Prolinks-fusion and STRING-fusion introduced fewer novel proteins for each link. Truong-fusion however behaved almost exactly the same as CODA, suggesting that this behaviour may be a feature of domain fusion methods.

It seems therefore that, for a given query protein, gene fusion methods provide more links to other proteins and thus increase the probability that there will be functional information available to annotate the query protein. This could be particularly important for query proteins from genomes with a low coverage of functional annotation. Where annotation is more frequent, domain fusion methods may provide a greater increase in coverage by identifying associations for more proteins. Ultimately this suggests that gene and domain fusion methods are complementary and can be used side by side.

#### Overlap between the results of different methods

**Figure 3:**
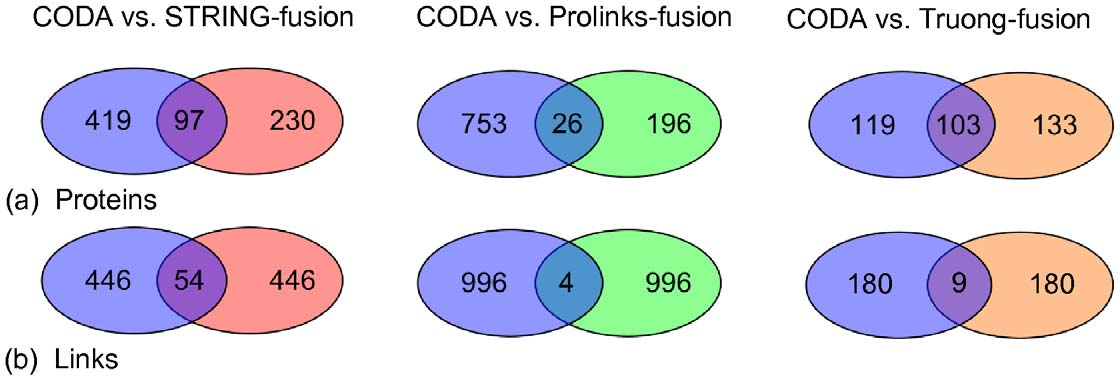
Overlap between the results of different methods. Overlap in (a) proteins involved in linkages and (b) linked pairs of proteins identified with yeast genome as query. Data is shown for the top scoring 500 hits for CODA and STRING-fusion, the first 1000 hits for CODA and Prolinks-fusion and the first 189 hits for CODA and Truong-fusion. CODA is represented by blue ellipses, STRING-fusion by red and Prolinks-fusion by green and Truong-fusion by orange.

Do different fusion methods tend to identify functional links between the same proteins? There was only a small overlap between CODA and the gene fusion methods (STRING-fusion and Prolinks-fusion) in the proteins identified as involved in fusion events (Figure 3a). There was a larger overlap between CODA and Truong-fusion as might be expected from their more similar methodologies. In terms of the specific pairwise associations found the overlap was however rather small (Figure 3b). Out of 500 predicted functional associations CODA and STRING-fusion shared only 54, with only 97 common proteins. CODA and Prolinks-fusion shared only four out of 1000 functional associations and 26 of the proteins. Despite their similar methodologies and genes identified in fusion events, CODA and Truong-fusion find only nine of the same functional links amongst the first 189 hits. These results further indicate that there is potential for integrating different methods of gene and domain fusion to increase the overall prediction power for determining proteins involved in common biological processes.

The reason for the low overlap between CODA and Truong-fusion is that, despite both using domains to identify fusions, they have rather different approaches for reducing false positives. For example, if in yeast, protein *A* contains domain *x* and protein *B* contains domain *y*. Both human and mouse genomes contain a gene with both *x* and *y* domains. If there are fewer than 10 pairs of genes in yeast with *x* or *y* domains, Truong's method will identify a functional linkage between *A* and *B*. In this case there is one gene with domain *x* and there are nine with domain *y*, making a total of nine possible linkages and Truong-fusion accepts this linkage. Truong does not take account of the number of copies in the target genome. In this case there are nine genes with domain *x* and 26 with domain *y* in mouse and even more in human. CODA therefore gives a score of 0.13 (1/(1+9)+1/(26+9)), which is not significant for functional similarity. CODA is designed to score the uncertainty about whether the fused domains and the domains being linked are orthologues; in this case it is clearly not certain. CODA and Truong often disagree because CODA takes into account the frequency of domains in the target genome (that containing the fusion gene), which Truong's method does not. The two methods agree on those linkages about which there is little or no uncertainty regarding orthology in the relationship between fused and unfused domains. They differ however when there is greater uncertainty.

The overlap between CODA and STRING-fusion is apparently higher than for CODA and Truong-fusion. STRING-fusion is strict about orthology when calling a fusion event (using bi-directional best hits) and this is, in theory, more similar to a high-scoring CODA hit than a high-scoring Truong-fusion hit.

Of the nine links identified by both Truong-fusion and CODA, eight are known from experiments to act in the same pathway. The ninth is either a novel result or a false positive (Thymidylate synthase and Protein VHS3). More generally, we found, using the full STRING database, that those links which overlapped between CODA and any of the other fusion methods tended to have evidence from other types of prediction methods, most often gene neighbourhood. This might be expected due to the similarity of gene/domain fusion and gene neighbourhood methods, compared to say co-expression, which leverages more distinct information. 70% of the CODA-Truong, 48% of the CODA-STRING and 50% of the CODA-Prolinks overlaps were also found by gene neighbourhood. This is compared to 30%, 17% and 25% for co-expression.

#### Assessment of performance on the human genome

In previous work the analysis of gene fusion for function prediction has been largely limited to prokaryotes and yeast. Detecting functional relationships between proteins using gene/domain fusion in higher eukaryotes is hampered by expanded gene/domain families. In order to examine this we have tested the performance of methods on the human genome.

**Figure 4:**
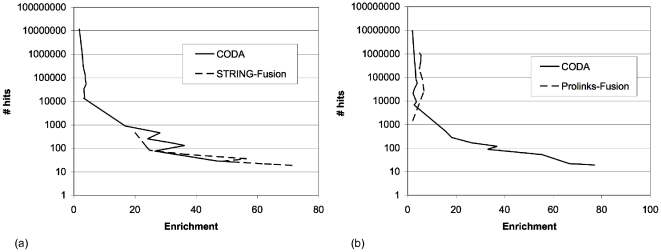
Comparisons between CODA and other methods on the human genome. (a) Relative performance of CODA and STRING-fusion methods on the STRING dataset. (b) Relative performance of CODA and Prolinks-fusion on the Prolinks dataset.

Figure 4a shows that STRING-fusion and CODA performed well despite the increased problems of promiscuity and large gene/domain families in the human genome. CODA found 3932 hits at an enrichment of 10. STRING-fusion found a maximum of 561 hits for an enrichment of ∼20; at this enrichment CODA found 1118 hits. STRING-fusion was able to achieve the highest enrichment of the two methods, finding 20 hits for an enrichment of 70.

Prolinks-fusion performed less well on the human dataset than in yeast, even when allowing homologous pairs (Figure 4b). In this dataset, CODA found 1611 protein pairs for an enrichment of 10, while Prolinks-fusion found none. The greatest enrichment that Prolinks-fusion achieved in human was 6.7, although it did find >25000 pairs at this level. At higher levels of enrichment CODA is able to find ∼100 hits for an enrichment of >30. Note that CODA finds fewer hits in the Prolinks dataset than the STRING dataset as the Prolinks dataset is somewhat smaller, containing 168 genomes versus 373 in STRING.

The Truong-fusion results had been obtained using Swiss-Prot release 39 and TrEMBL release 17. These datasets were released in 2001 at which point the human genome was not complete. CODA requires complete genomes for accurate scoring and therefore it was not possible to compare against Truong-fusion for human. However Truong-fusion managed to find 235 associations between human proteins for an enrichment of 28.

### Applying CODA to identify associations between proteins

Annotation from the OMIM (Online Mendelian Inheritance in Man) database was mapped to all associations identified by CODA in human using Gene3D [14]. Only those links identified by CODA with a score of 0.56 or greater were included. This score cut-off was found to represent an enrichment of 10 for both yeast and human datasets. We identified uncharacterised proteins, which were linked directly to proteins involved in human disease.

The uncharacterised human protein Q6NZ37 (UniProt accession) was found to be associated with several proteins involved in mental disorders. TPH2 (Q8IWU9) is known to be involved in major depressive disorder (MIM:608516) and is directly involved in the biosynthesis of serotonin from L-tryptophan. Another associate of Q6NZ37, TPH (P17752; MIM:191060) has been shown to be involved in suicidal behaviour, thought to be related to depression [15]. Several other associates of Q6NZ37 are known or thought to be involved in serotonin biosynthesis. Additional associates sialic acid synthase (NANS; Q9NR45) and quinolinate phosphoribosyltransferase (QPRT; Q96G22) are known to be involved in brain function. Sialic acid is linked with development of neural tissues during embryogenesis [16] and quinolate levels in human brain are thought to be involved in the pathogenesis of neurological disorders (MIM: 606248). Quinolate metabolism also feeds into serotonin metabolism. Another example of a functionally coherent network of interactions identified by CODA centred on DNA ligase I. Mutations in this gene have been linked with rare cases of multi-symptomatic disease [17]. A protein of unknown function, Q96LW4, is linked to this and other known ligases, suggesting that it too is involved in DNA replication and potentially forms of multi-symptomatic disease. Searches within STRING, Prolinks and Truong data gave no associations for either of these proteins.

### Additional functional coverage produced by CODA

We wanted to determine how much additional functional coverage of the human genome could be generated by CODA. To do this we considered those proteins between which CODA found high confidence functional associations and asked how many could, based on these associations, be assigned a GO term where before they were unannotated. CODA found 1453 high confidence (CODA score > = 0.56) associations between 900 human proteins using the Gene3D dataset. Of these 900 proteins, 664 could already be annotated with a GO biological process term using annotation from GOA (Camon et al., 2004) and allowing all evidence types. Of the remaining 236 unannotated proteins, 107 could be annotated by transferring high quality GO annotation (experimental evidence and author statements) using the associations established by CODA. Although this is a small number of proteins in terms of the whole human genome, these proteins have not previously been annotated with GO terms. The annotations for these proteins are presented as Supplementary Dataset S1.

## Discussion

We present a new domain fusion method designed to be accurate in predicting functional associations between proteins in higher eukaryotes. The genomes of higher eukaryotes contain large protein domain families, which make the detection of functional associations by gene/domain fusion less reliable. To cope with this problem, previous methods have either considered only orthologues [9] or implemented a scoring system based on the frequencies of domain families in the whole target sequence database [7]. We have implemented a scoring system, but rather than using counts of domain frequency across all genomes as in previous scoring methods the CODA score uses domain counts within individual genomes. This reduces the problem of large domain families and allows good scores between pairs where there is a genome in which the fusion protein has few homologues. CODA was shown to cope well with the problem of large, promiscuous domains families.

Gene/domain fusion methods in general have been thought to perform better in prokaryotes than eukaryotes as prokaryotes tend to have smaller families of homologous genes/domains [7]. Here it was shown that CODA, STRING-fusion and Truong-fusion are both robust to the complexities of the human genome, achieving high accuracy and coverage, with CODA finding ∼7 times more results than STRING for a reasonable error rate. However, at very low error rates STRING outperformed CODA. Prolinks-fusion did not perform as well in human as in yeast, possibly due to the increased problems of large homologous domain families and promiscuous domains.

These methods seem to occupy two distinct niches. The methods which can achieve the highest accuracy but which provide a relatively small number of hits (STRING-fusion and Truong-fusion) are useful for identifying high quality sets of associations. However for any particular protein it is unlikely that they will find an association. Methods such as Prolinks-fusion and CODA can provide less certain associations for a greater number of hits and therefore would be more appropriate where the other methods cannot provide associations.

Interestingly there is little overlap between the methods in terms of the functional links they predict and even the proteins included in the links. This suggests that the particular implementation greatly affects the links obtained (e.g. using domains vs. whole proteins). Furthermore, much as different genome context methods have been combined to produce larger sets of confident predictions (e.g. [3]), using different implementations of the gene/domain fusion method could allow a greater number of predictions overall.

Finally it was shown that CODA is able to identify putative disease-related proteins in humans. Furthermore, many previously unannotated human proteins were assigned GO terms using CODA suggesting that this approach will also be able to annotate previously unannotated proteins in many other genomes. These results are provided as a supplementary Dataset S1.

CODA is available for download and can be used to determine functional relationships between proteins in any genome of interest to the user. Pre-calculated results are also available via web services.

## Materials and Methods

### Gene3D Multi-Domain Architecture datasets

Co-Occurrence of Domains Analysis (CODA) requires Multi-Domain Architectures (MDAs) of proteins for complete genomes. An MDA is a symbolic representation of the predicted domains for a protein. The order and frequency of domains in a protein is not considered by CODA and so discontinuous domains can be collapsed and repeats ignored.

Several alternative MDA datasets were generated using domain assignments from Gene3D v5 [14], each covering 527 complete genomes (50 eukaryotes, 438 eubacteria and 39 archaea). Datasets contained either only CATH domains, only Pfam domains or a combination of the two. Further details of the construction of these datasets are presented in Figure S6 and Table S1. These datasets were used in developing the CODA method.

Only the results of the methods against which we compare CODA were available, rather than the algorithms themselves. Therefore, in order to compare CODA against these methods, it was necessary to run CODA on the datasets used to generate those results. For STRING and Prolinks, descriptions of the sequences used are available from the respective web-servers. The Truong dataset combined Swiss-Prot release 39 and TrEMBL release 17. The Swiss-Prot release was retrieved from the EBI FTP server (ftp://ftp.ebi.ac.uk/pub/databases/swissprot/sw_old_releases), while TrEMBL release 17 was kindly provided by the PANDA group at the EBI.

All STRING and Prolinks sequences were scanned with Pfam HMMs using the same pfam_scan.pl protocol used for Gene3D [18]. Details of these datasets are shown in Supplementary Table S2.

Truong-fusion and CODA both use Pfam domains, so in order to avoid giving CODA an advantage by using the most recent Pfam annotation, the Truong dataset was annotated using Pfam domain annotation from the contemporary Swiss-Prot and TrEMBL records. The STRING and Prolinks datasets comprise protein sequences from completed genomes. The Truong dataset however gave no information of which genomes in the dataset were complete and it is difficult to determine which genomes were completed at this time. Therefore those proteins from species which currently remain unsequenced were removed. The result is that some incomplete genomes will remain and this may reduce the performance of CODA, which expects complete genome information to accurately score its results.

### A benchmark for functional similarity using Gene Ontology terms

The aim of the CODA method is to identify pairs of proteins which are involved in similar biological processes. In order to benchmark CODA it was therefore necessary to determine the functional similarity between an arbitrary pair of proteins. We compared the biological process Gene Ontology (GO) terms of proteins using the semantic similarity approach of Resnik et al. [19], [20] and GO annotation from Gene3D. This approach requires a corpus of terms in calculating its statistics and this was varied according to whether the benchmark was performed in yeast or human and whether the dataset was Gene3D, STRING, Prolinks or Truong. Those terms with evidence type ‘Inferred from Electronic Annotation (IEA)’, ‘No biological Data available (ND)’ and ‘Inferred from Genomic Context (IGC)’ were removed to avoid the circularity of benchmarking a method using results derived from similar methods. The coverage of each of these datasets by relevant GO terms is shown in Supplementary Table S3. The GOSS score between any two proteins was taken as the maximum GOSS score between any pair of terms associated with those proteins.

In this benchmark false positives could not be directly determined as many proteins were unannotated or annotated with relatively non-specific GO terms. Therefore, instead of precision we calculated enrichment, based on the number of positive hits expected by chance. Protein pairs identified by a method, which exceeded a GOSS score of 4, were considered true positive hits. Only ∼3% of GOSS scores were > = 4 (see Figure S7). For both human and yeast datasets, GOSS scores of 4 and above are sufficiently rare that they are unlikely to be picked by random chance (<1 in 20). Considering all protein pairs in yeast (i.e. including those with no appropriate GO terms), the likelihood of a score > = 4 was 0.0167. From 50 random protein pairs, we would therefore expect to see 0.835 (50×0.0167) functionally similar pairs. If 10/50 pairs predicted by CODA have a GOSS score > = 4, CODA has achieved an enrichment of 11.98 (10 observed true positives divided by 0.835 expected true positives). The distribution of GOSS scores for the human genome was very similar to the yeast genome, although 93.7% of pairs did not have a GOSS score. For the human genome ∼3% of GOSS scores were > = 4. STRING, Prolinks and Truong datasets also had <5% GOSS scores > = 4. The proportion of expected positives used in calculating enrichment was varied appropriately for each dataset.

### The CODA score

Co-Occurrence of Domains Analysis (CODA) uses a Multi-Domain Architecture (MDA) representation of proteins in complete genomes (target genomes) to discover pairs of proteins involved in common biological processes within a complete genome of interest (the query genome). It is a novel approach in the domain fusion idiom using a new scoring method.

Here we consider how the method is implemented for a particular pair of proteins *i* = (*p*,*q*) in a query genome *g*. *P* is the set of domains in protein *p*. *a* 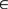 *P* denotes that protein *p* contains a domain of superfamily *a*. *J* is the set of domain pairs *j* = (*a*,*b*) where *a* 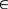 *P*, *b* 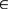 *Q*. In other words *J* consists of all the distinct pairs of domains between proteins *p* and *q*. It is also required that *P*∩*Q* = {}, as the two proteins must not share any domains of the same family.

To determine a fusion event we require that a target genome (one other than the query genome) contains a protein *s* where *a* 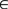 *S* and *b* 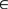 *S*, i.e. domains which are separated in the query genome are found fused in the target genome. The set *T* comprises those genomes other than *g*, which contain such proteins *s*. For a domain pair *j* in genome *g*, the fusion score *C_j_* is taken as a maximum over all genomes in *T*:
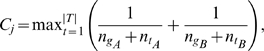
(1)where |*T*| is the number of elements of set *T* (i.e. the number of target genomes), n_gA_ and n_gB_ are the frequencies of domain *A* and domain *B* respectively in genome *g* and n_tA_ and n_tB_ are the frequencies of domains A and B respectively in genome t.

For a particular protein pair *i* in query genome *g*, the maximum *C_j_* is taken over all possible domain pairs *j*.
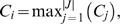
(2)where |*J*| is the number of elements in set *J* (i.e. distinct domain pairs). Thus *C_i_* is the CODA score for proteins *p*,*q* (pair *i*); the best (highest) score over all domain pairs between the proteins and over potential fusion proteins in all genomes (other than the query genome). The important novel aspect of this score is that it takes the maximum score over all the genomes whereas other methods do not consider target genomes individually. The score was chosen to reflect the uncertainty that fused domains and their unfused relatives are orthologues. The highest (best) possible score is 1. This occurs when there is only one example of each domain family in the query genome and one fused protein in a target genome, with no other domain homologues. In this case it is highly likely that the query protein domains are orthologous to the target protein. Several other scoring schemes were trialled, including that used by Marcotte & Marcotte [7], however the CODA score presented above was found to be most accurate for domain fusion.

## Supporting Information

Figure S1 Comparative performance of Pfam, Pfam-CATH, CATH and CATH-Pfam MDA datasets on the yeast genome. Enrichment is the ratio of true positives achieved by CODA to the number expected by chance. Points are plotted at successive score cut-offs. At an enrichment of 10, Pfam-CATH performed best with 1791 hits; Pfam achieved 1663 hits, CATH-Pfam 792 and CATH 296. At higher enrichment (e.g. 15), the Pfam dataset outperforms all others and finds ∼500 hits. Datasets based principally on CATH domains (CATH-Pfam and CATH) performed less well than those based on Pfam domains. This may be because CATH superfamilies tend to be broader than Pfam families, including more functional subfamilies. This could result in generally reduced scores for hits involving these larger families. Additional analysis using CATH subfamilies did not improve performance however (Figure S1) and thus it is more likely that CATH had lower performance due to lower coverage of the genomes. Pfam MDA datasets were chosen over Pfam-CATH due to a similar performance at moderate enrichment and superior performance at higher enrichment. CODA should be used with a score cut-off of 0.56 to achieve an enrichment of 10 on this dataset and 0.65 for an enrichment of 15.
(0.60 MB EPS)

Figure S2 Performance of CODA using CATH domains, with and without subfamilies. CATH domains showed lower performance than Pfam domains in detecting functional relationships between proteins using CODA. This could have been due to low coverage of CATH domains relative to Pfam or because CATH has larger families causing low scores for many hits. CATH superfamilies were clustered at varying sequence identity cut-offs (30, 35, 40, 50, 60, 70, 80, 90, 95 and 100%) using an in-house implementation of directed multi-linkage clustering. The domain counts used in the CODA score were then adjusted using these clusters. Let us say that there are two proteins in yeast, each with one domain. The first protein contains domain A and the second domain B. A protein is found in *E. coli* which is a fusion of these two domains: A′B′. Let us say that A and A′ are in the same 50% cluster but not the same 60% cluster, i.e. they share 50% sequence identity. The counts for ngA in the CODA score (equation 1) then only include the number of members of the same 50% cluster that belong to yeast. ngB is the number of members of that 50% cluster which belong to *E. coli*. Likewise, if B and B′ are in the same 70% cluster but not the same 80% clusters, then the counts are taken from that 70% cluster. Using subfamilies slightly improves performance at high enrichment but only where there are few hits. We therefore concluded that the reduced performance of CATH relative to Pfam was related to a mixture of lower coverage of genomes and than the size and functional specificity of the families.
(0.52 MB EPS)

Figure S3 Relationship between number of links and proteins for first 500 novel links between CODA (blue) and STRING-fusion (red).
(0.55 MB EPS)

Figure S4 Relationship between number of links and proteins for first 1000 novel links between CODA (blue) and Prolinks-fusion (green).
(0.57 MB EPS)

Figure S5 Relationship between number of links and proteins for first links between CODA (blue) and Truong-fusion (orange).
(0.56 MB EPS)

Figure S6 Overlap criterion for combining CATH and Pfam domains into a single dataset. The datasets which included both CATH and Pfam domains were generated in two ways. The CATH-Pfam dataset had CATH domains assigned first, while Pfam-CATH had Pfam domains assigned first. Examples of the second type of domain were added if the overlap between them and the already assigned, primary domains was no greater than 30% in both directions (see Figure S1). The initial set of CATH domains did not overlap with each other, nor did the Pfam domains. This resulted in 4 different datasets – CATH, Pfam, CATH-Pfam and Pfam-CATH. Table S1 gives details on the domain coverage of these datasets. Existing datasets of CATH or Pfam domains do not overlap within themselves. When CATH and Pfam are combined there are frequent overlaps as many domains are equivalent between the datasets and criteria for domain boundaries vary. Shown in Figure S6 is an example for the CATH-Pfam dataset, where CATH domains are placed first. The percentage of residues of either domain involved in the overlap must not exceed 30%.
(0.14 MB TIF)

Figure S7 Distribution of biological process GOSS scores between yeast proteins in the Gene3D dataset. Proteins without appropriate GO terms were excluded. GOSS score bins were bounded such that the bin labelled 2 contains values > = 2 and <2.5. The bars represent the frequency and the blue line represents the cumulative proportion of GOSS scores which have less than the stated value. We show here the distribution of GOSS scores in the yeast genome. Protein pairs which score 0 because they do not have comparable GO terms were ignored (43.4% of all yeast pairs).
(0.63 MB EPS)

Table S1 Size of Gene3D datasets and genome coverage with different Multi-Domain Architecture (MDA) types. Coverage is calculated as the percentage of proteins which have at least one domain. The CATH-Pfam and Pfam-CATH datasets therefore appear identical, although their domain assignments are not.
(0.03 MB DOC)

Table S2 Coverage of STRING, Prolinks and Truong datasets with Pfam domains. Coverage is calculated as the percentage of proteins with at least one domain. Raw numbers are shown in brackets.
(0.03 MB DOC)

Table S3 Percentage of individual protein for each genome in each dataset which has at least one relevant GO term.
(0.03 MB DOC)

Dataset S1 Novel functional annotation assigned to human gene products using CODA.
(0.33 MB XLS)
