# Supplementary figures and images for: CODA: Accurate Detection of Functional Associations between Proteins in Eukaryotic Genomes Using Domain Fusion
Source: PLoS One. 2010 Jun 1;5(6):e10908. doi: 10.1371/journal.pone.0010908 (PMC2879367; doi:10.1371/journal.pone.0010908)

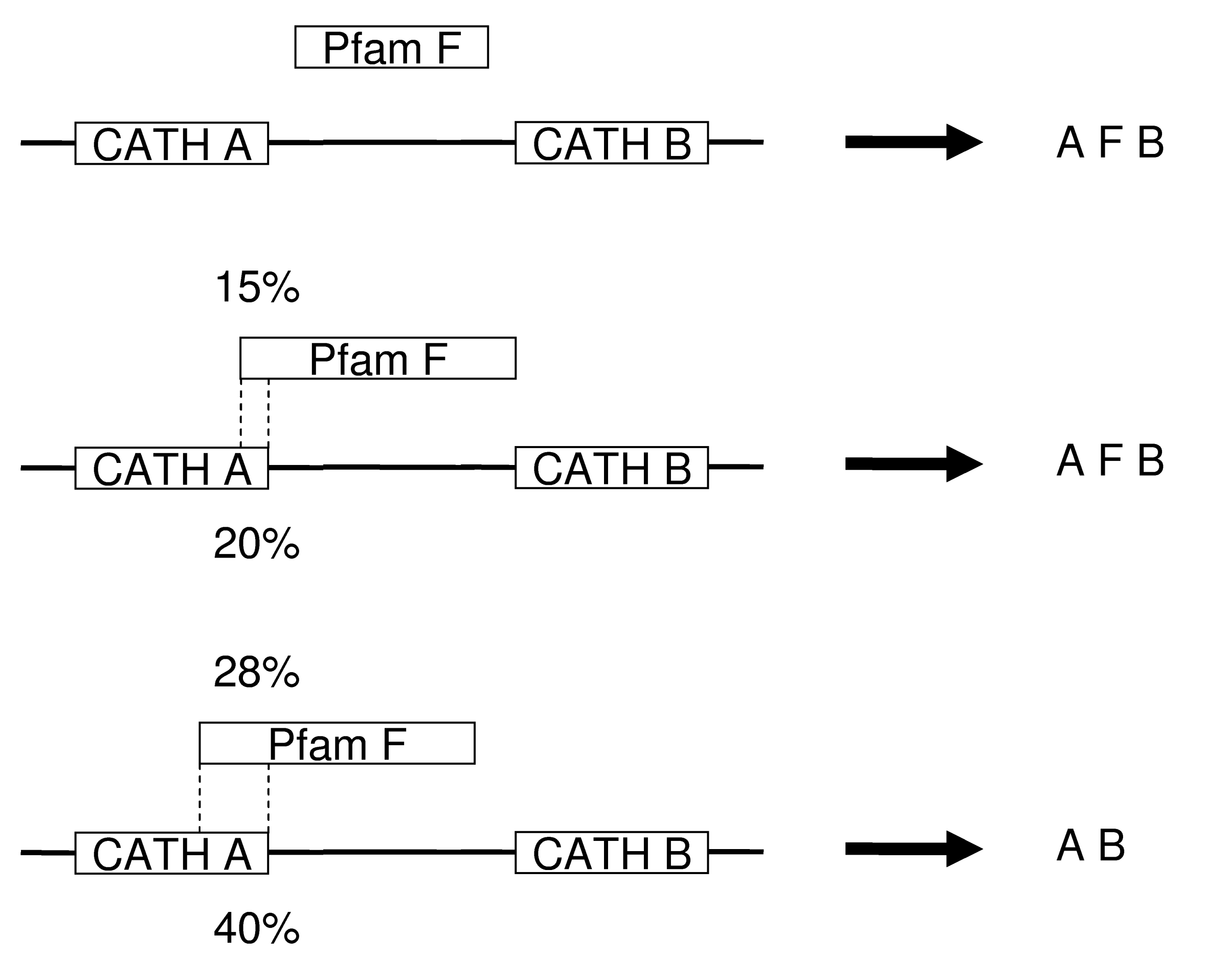

Supplement: Figure S6 — Overlap criterion for combining CATH and Pfam domains into a single dataset. The datasets which included both CATH and Pfam domains were generated in two ways. The CATH-Pfam dataset had CATH domains assigned first, while Pfam-CATH had Pfam domains assigned first. Examples of the second type of domain were added if the overlap between them and the already assigned, primary domains was no greater than 30% in both directions (see Figure S1). The initial set of CATH domains did not overlap with each other, nor did the Pfam domains. This resulted in 4 different datasets – CATH, Pfam, CATH-Pfam and Pfam-CATH. Table S1 gives details on the domain coverage of these datasets. Existing datasets of CATH or Pfam domains do not overlap within themselves. When CATH and Pfam are combined there are frequent overlaps as many domains are equivalent between the datasets and criteria for domain boundaries vary. Shown in Figure S6 is an example for the CATH-Pfam dataset, where CATH domains are placed first. The percentage of residues of either domain involved in the overlap must not exceed 30%. (0.14 MB TIF) [file pone.0010908.s006.tif]
