# Supplementary material for: CODA: Accurate Detection of Functional Associations between Proteins in Eukaryotic Genomes Using Domain Fusion
Source: PLoS One. 2010 Jun 1;5(6):e10908. doi: 10.1371/journal.pone.0010908 (PMC2879367; doi:10.1371/journal.pone.0010908)
Supplement: Table S1 — Size of Gene3D datasets and genome coverage with different Multi-Domain Architecture (MDA) types. Coverage is calculated as the percentage of proteins which have at least one domain. The CATH-Pfam and Pfam-CATH datasets therefore appear identical, although their domain assignments are not. (0.03 MB DOC) [file pone.0010908.s008.doc]

| Dataset | Total proteins | Yeast coverage (of 5586 distinct protein sequences) | Human coverage (of 34888) |
| --- | --- | --- | --- |
| CATH | 821801 | 38% (2130) | 40% (13831) |
| Pfam | 1423060 | 73% (4050) | 65% (22736) |
| CATH-Pfam | 1495200 | 76% (4226) | 68%(23679) |
| Pfam-CATH | 1495200 | 76% (4226) | 68% (23679) |
