# Supplementary material for: CODA: Accurate Detection of Functional Associations between Proteins in Eukaryotic Genomes Using Domain Fusion
Source: PLoS One. 2010 Jun 1;5(6):e10908. doi: 10.1371/journal.pone.0010908 (PMC2879367; doi:10.1371/journal.pone.0010908)
Supplement: Table S2 — Coverage of STRING, Prolinks and Truong datasets with Pfam domains. Coverage is calculated as the percentage of proteins with at least one domain. Raw numbers are shown in brackets. (0.03 MB DOC) [file pone.0010908.s009.doc]

| Resource | Genomes | Total dataset coverage by Pfam domains | Yeast Pfam coverage | Human Pfam coverage |
| --- | --- | --- | --- | --- |
| STRING v7 | 373 | 71% (1074952/1513782) | 64% (4245/6680) | 74% (16371/22218) |
| Prolinks v2.0 | 168 | 73% (429173/590444) | 73% (4195/5761) | 74% (17266/23213) |
| Truong dataset | 210 | 50%  (128332/257962) | 44%  (2935/6690) | n/a |
