# Supplementary material for: CODA: Accurate Detection of Functional Associations between Proteins in Eukaryotic Genomes Using Domain Fusion
Source: PLoS One. 2010 Jun 1;5(6):e10908. doi: 10.1371/journal.pone.0010908 (PMC2879367; doi:10.1371/journal.pone.0010908)
Supplement: Table S3 — Percentage of individual protein for each genome in each dataset which has at least one relevant GO term. (0.03 MB DOC) [file pone.0010908.s010.doc]

| Dataset | Yeast | Human |
| --- | --- | --- |
| Gene3D v6 | 75% (4203/5586) | 18% (6192/34888) |
| STRING v7 | 67% (4447/6680) | 22% (4861/22218) |
| Prolinks v2.0 | 76% (4385/5761) | 23% (4980/23213) |
| Truong dataset | 58%  (3885/6690) | n/a |
